# Supplementary material for: The associations between Schistosoma mansoni infection, pre-treatment symptoms, praziquantel side effects, and treatment efficacy in Ugandan school-aged children
Source: PLoS Negl Trop Dis. 2025 Oct 9;19(10):e0013167. doi: 10.1371/journal.pntd.0013167 (PMC12533968; doi:10.1371/journal.pntd.0013167)
Supplement: S1 Table — (DOCX) [file pntd.0013167.s001.docx]

**S1 Table.** The number of students in each school participating in the research in 2004.

|  | Number of tested students | Number of students included in analysis |
| --- | --- | --- |
| Bugoto LV | 123 | 94 |
| Musubi CoG | 68 | 68 |
| Total | 191 | 162 |

At baseline in Bugoto Lake View (LV) primary school 123 children, and in Musubi Church of God (CoG) primary school 68 children were recruited into the study. From Bugoto LV, only 94 children provided follow-up data and all of the children in Musubi CoG provided follow-up data, such that the sample sizes used in the HMM were 94 for Bugoto LV and 68 for Musubi CoG.
