# Supplementary material for: The associations between Schistosoma mansoni infection, pre-treatment symptoms, praziquantel side effects, and treatment efficacy in Ugandan school-aged children
Source: PLoS Negl Trop Dis. 2025 Oct 9;19(10):e0013167. doi: 10.1371/journal.pntd.0013167 (PMC12533968; doi:10.1371/journal.pntd.0013167)
Supplement: S1 Fig — Note the jittering of points to prevent them overlapping. (DOCX) [file pntd.0013167.s002.docx]

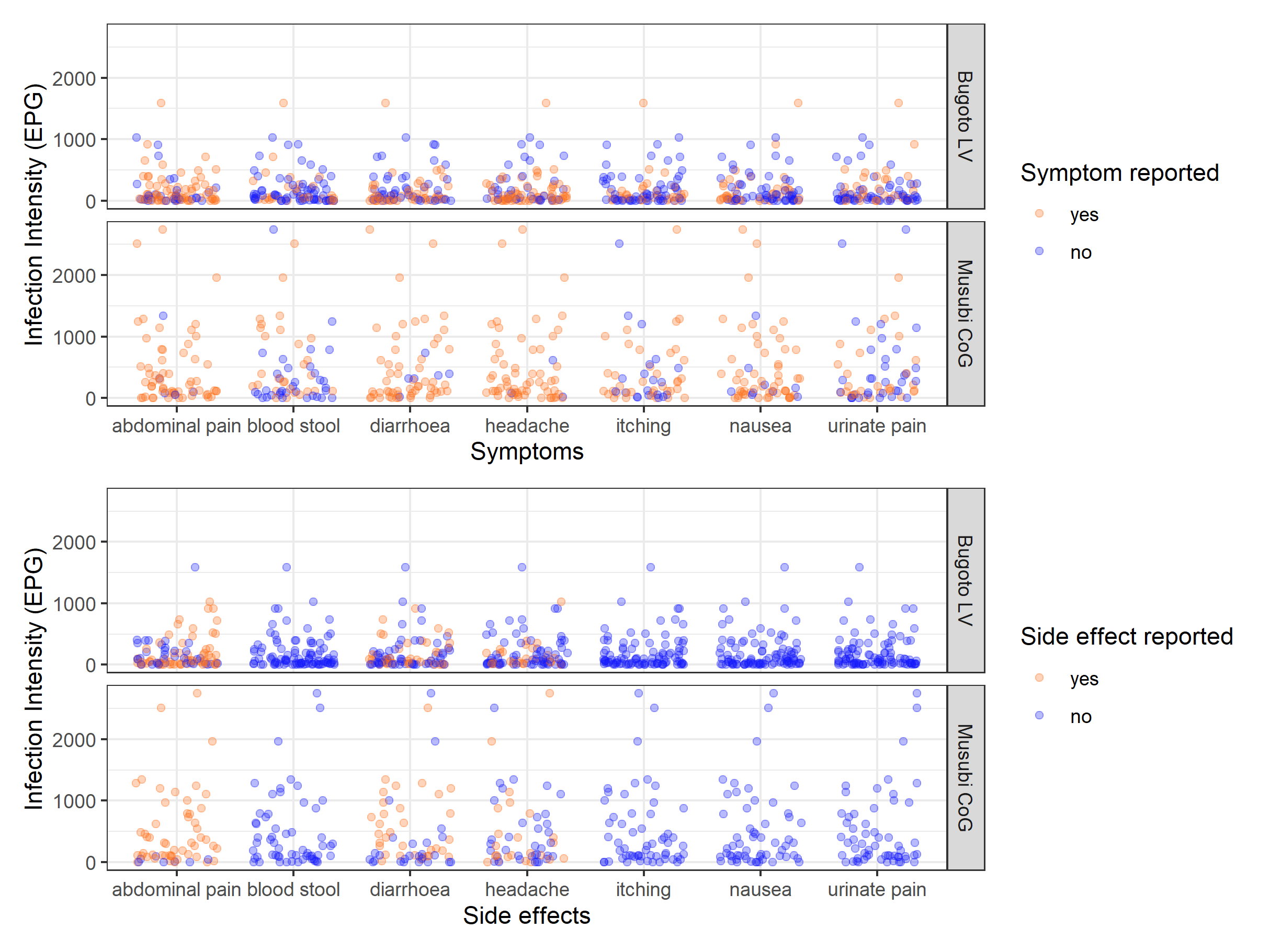


**S1 Fig.**  The *Schistosoma mansoni* infection intensity of every child in Bugoto Lake View (LV) (top graph in both panel) and Musubi Church of God (CoG) (bottom graph in both panel) primary schools who reported (orange), or did not report (blue) pre-treatment symptoms (x-axis of top panel) or post-treatment side effects (x-axis of bottom panel) in 2004. Note the jittering of points to prevent them overlaying one another.
