# Supplementary material for: The associations between Schistosoma mansoni infection, pre-treatment symptoms, praziquantel side effects, and treatment efficacy in Ugandan school-aged children
Source: PLoS Negl Trop Dis. 2025 Oct 9;19(10):e0013167. doi: 10.1371/journal.pntd.0013167 (PMC12533968; doi:10.1371/journal.pntd.0013167)
Supplement: S2 Fig — (DOCX) [file pntd.0013167.s003.docx]

**
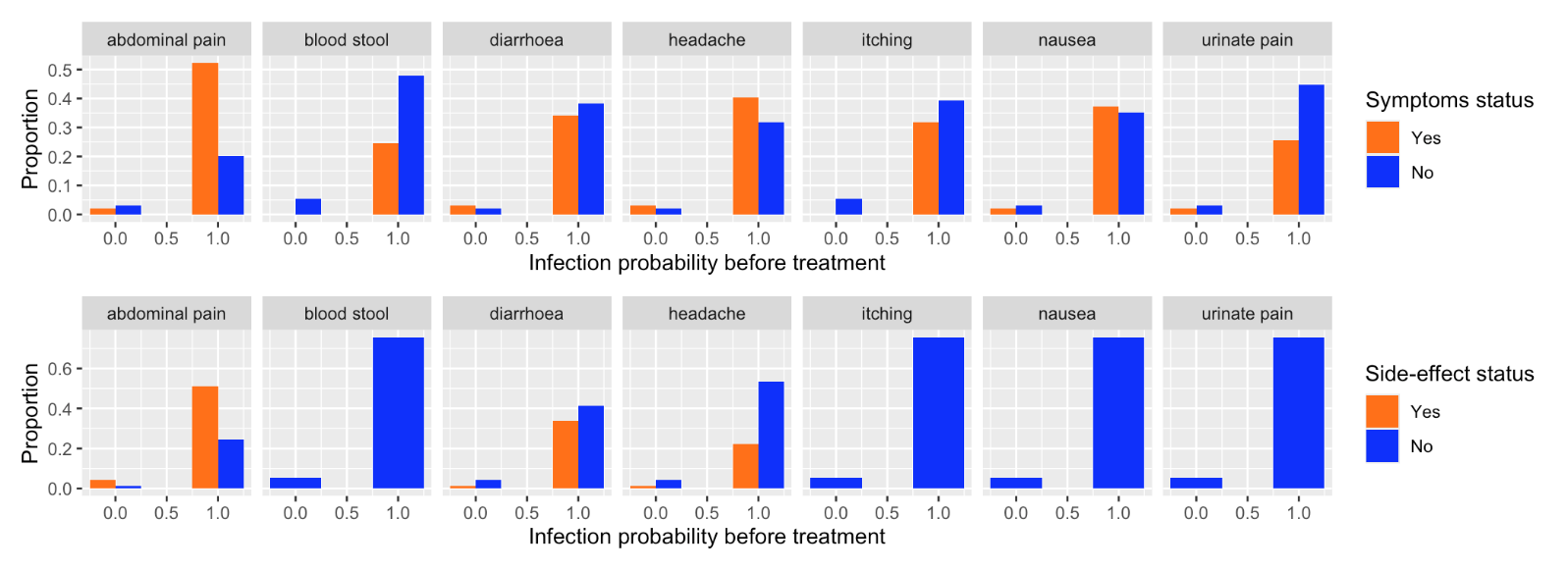
**

**S2 Fig.** The proportion of Bugoto Lake View (LV) primary school children who reported (orange), or did not report (blue) pre-treatment symptoms (top row) and post-treatment side effects (bottom row) compared with *Schistosoma mansoni* infection probability.

**Bugoto LV**

**Symptoms**

Half or fewer infected students reported blood-in-stool (24.5%), diarrhoea (34.0%), headache (40.4%), itching/rash (31.9%), or nausea (37.2%) as symptoms. However, 52.1% of infected students reported experiencing abdominal pain before treatment. (Figure S2).

**Side effects**

After treatment, 34.0% of infected students reported diarrhoea and 22.3% reported headache as side effects, and more than half of students who were infected reported that they experienced abdominal pain. There was no student that reported itching, urinating pain, nausea, and blood stool as side effects.
