# Supplementary material for: The associations between Schistosoma mansoni infection, pre-treatment symptoms, praziquantel side effects, and treatment efficacy in Ugandan school-aged children
Source: PLoS Negl Trop Dis. 2025 Oct 9;19(10):e0013167. doi: 10.1371/journal.pntd.0013167 (PMC12533968; doi:10.1371/journal.pntd.0013167)
Supplement: S3 Fig — (DOCX) [file pntd.0013167.s004.docx]

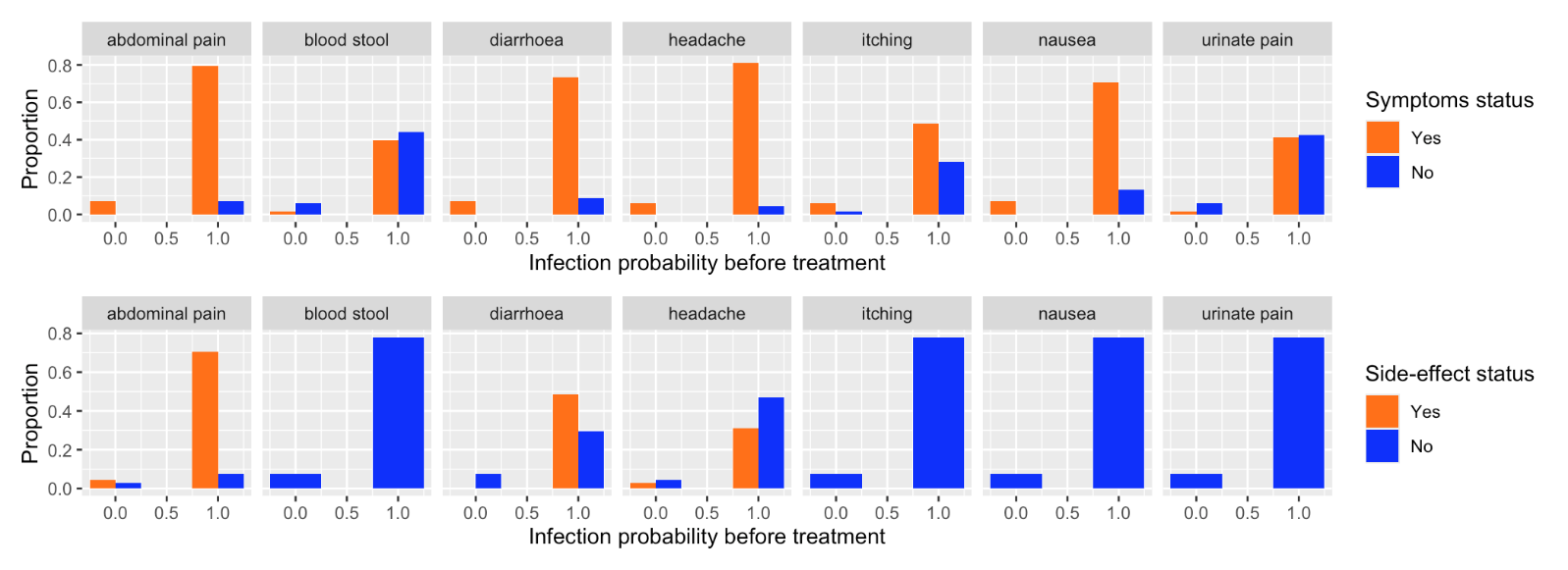


**S3 Fig.** The proportion of Musubi Church of God (CoG) primary school children who reported (orange), or did not report (blue) pre-treatment symptoms (top row) and post-treatment side effects (bottom row) compared with *Schistosoma mansoni* infection probability.

**Musubi CoG**

**Symptoms**: Infected students reported pre-treatment symptoms of abdominal pain (79.4%), diarrhoea (73.5%), headache (80.9%), nausea (70.6%). 48.5% of students who were infected reported that they experienced itching/rash, and among infected students, the proportion of students who reported blood-in-stool (39.7%) and pain-when-urinating pain (41.2%) was comparable to those who did not report them (blood-in-stool: 44.1%, pain-when-urinating: 42.6%) (Figure S3).

**Side effects**: After treatment, there was no student reporting blood-in-stool, itching/rash, nausea, and pain-when-urinating as side effects. Among infected students, 70.6% of students reported abdominal pain, and the proportions of students who reported diarrhoea (48.5%) and headache (30.9%) as side effects were decreased by 25% and 50% respectively.
