# Supplementary material for: The associations between Schistosoma mansoni infection, pre-treatment symptoms, praziquantel side effects, and treatment efficacy in Ugandan school-aged children
Source: PLoS Negl Trop Dis. 2025 Oct 9;19(10):e0013167. doi: 10.1371/journal.pntd.0013167 (PMC12533968; doi:10.1371/journal.pntd.0013167)
Supplement: S4 Fig — The size of dots means number of students. (DOCX) [file pntd.0013167.s005.docx]

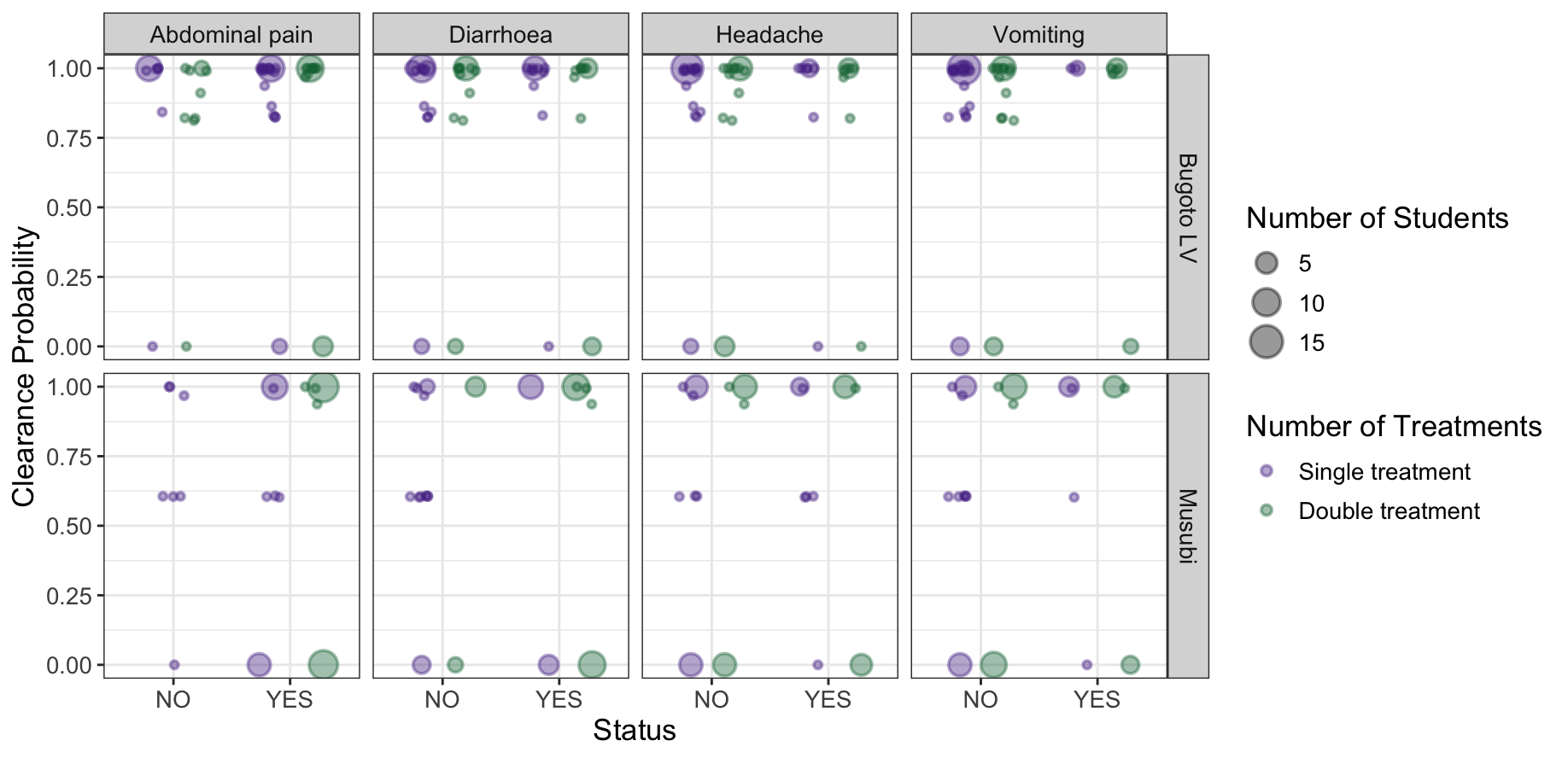


**S4 Fig.** The clearance probability compared with status of self-reported side effects (abdominal pain, diarrhoea, headache, and vomiting) after single (dark purple) or double (dark green) praziquantel treatment in Bugoto Lake View and Musubi Church of God primary schools, in 2004. The size of dots reflects the number of students.
