# Supplementary material for: The associations between Schistosoma mansoni infection, pre-treatment symptoms, praziquantel side effects, and treatment efficacy in Ugandan school-aged children
Source: PLoS Negl Trop Dis. 2025 Oct 9;19(10):e0013167. doi: 10.1371/journal.pntd.0013167 (PMC12533968; doi:10.1371/journal.pntd.0013167)
Supplement: Attachment — Submitted filename: Response to Reviewers_SideEffects_Yuetal.pdf [file pntd.0013167.s007.pdf]

University of Glasgow
Graham Kerr
University Avenue
Glasgow
G12 8QQ

August 26th, 2025

Re: Responses to reviewers' comments on manuscript PNTD-D-25-00778

Dear Dr Diemert,

Thank you to the editorial and review team for providing insightful comments. We have now addressed these, and we now hope this improved manuscript meets the standards for publication in PLoS Neglected Tropical Diseases.

Please find below, each comment addressed in detail, highlighting the changes we have made accordingly. The line numbers correspond to the Tracked Changes PDF document, due to the potential for formatting inconsistencies in the Tracked Changes Microsoft Word document.

Editor comments

Please ensure that the CRediT author contributions listed for every co-author are completed accurately and in full.
At this stage, the following Authors/Authors require contributions: Huanghehui Yu, Moses Arinaitwe, Adriko Moses, Narcis Kabatereine, Edridah M. Tukahebwa, David W. Oguttu, Aidah Wamboko, Annet Namukuta, Annet Enzaru, Joaquin M. Prada, Alan Fenwick, Joanne P. Webster, Poppy H. L. Lamberton, and Jessica Clark. Please ensure that the full contributions of each author are acknowledged in the "Add/Edit/Remove Authors" section of our submission form.
The list of CRediT author contributions may be found here: https://journals.plos.org/plosntds/s/authorship#loc-author-contributions

This has now been completed. As there is no indication in the online guidance or the portal, we have added this information to the bottom of the manuscript, after the acknowledgements.

We have noticed that you cited Figure S5 on page 11 line 236 in your manuscript. However, there is no corresponding file uploaded to the submission. Please upload

it as a separate file with the item type 'Supporting Information'. Or, if the figure is no longer to be included as part of the submission please remove all reference to it within the text.

We have searched for reference to an S5 in the main text, but cannot find this anywhere, including where indicated. The supplementary material has since been updated due to reviewer comments and we have checked that all figures are referenced in the main text.

We have noticed that you have uploaded Supporting Information files, but you have not included a list of legends. Please add a full list of legends for your Supporting Information files after the references list.

The supplementary figure legends are now provided at the end of the main manuscript text, after the references, as requested.

Please amend your detailed Financial Disclosure statement. This is published with the article. It must therefore be completed in full sentences and contain the exact wording you wish to be published.
1) State what role the funders took in the study. If the funders had no role in your study, please state: "The funders had no role in study design, data collection and analysis, decision to publish, or preparation of the manuscript."
2) If any authors received a salary from any of your funders, please state which authors and which funders.

The statement now reflects that the funders played no role in the study design, data collection and analysis, decision to publish, or preparation of the manuscript.

7) Please ensure that the funders and grant numbers match between the Financial Disclosure field and the Funding Information tab in your submission form. Note that the funders must be provided in the same order in both places as well. Currently, the order of the funders is different in both places. In addition, this grant "EPX0270821" is missing from the Financial Disclosure field.

This has now been rectified.

Reviewer 1's Comments:

Methods:

Line 170-172, Please add the batch number of the POC-CCA test that was used. Was POC-CCA testing done on fresh urines in 2004, or retrospectively (after storage)?

The POC-CCA testing was performed on fresh urine samples collected at each timepoint

in 2004, immediately after sample collection. This information has now been amended in the Methods section for clarity (lines 204-205). Batch numbers were unfortunately not recorded; however, the samples were processed using the original POC-CCA prototype with two drops of a buffer solution added after urine addition. This occurred prior to the batch-to-batch variation issues that began to occur when this buffer step was removed in the second prototype.

Results:

Line 294, Table 1:
- Please adjust the decimals, now some percentages do not have decimals while others have 2 decimals (1 decimal would be sufficient). The same for the text (in line 284 no decimal, while in the subsequent paragraph 2 decimals are used).

Thank you for highlighting this. We have now standardised the formatting of all percentages across both the text and Table 1 to display one decimal place for consistency and clarity.

- Can you specify whether the symptoms pre-treatment were ongoing (if they had them presently) or if they had ever experienced them? (same question for the text in line 286-289).

We agree that this may not have been sufficiently clear in the original text. The data used to describe pre-treatment symptoms refer to whether participants had ever experienced these symptoms prior to treatment. To avoid misunderstanding, we have now revised the sentence in the Methods section (line 231) to clarify this.

Line 315-316, In Table 2 you also include headache as symptom pre-treatment, but this is not mentioned in the text?

We thank the reviewer for pointing this out. We have now included mention of headache as a pre-treatment symptom in the main text to ensure consistency with Table 2, in line 347.

Line 314-327, please be clear in the text whether you talk about pre-treatment symptoms or post-treatment side effects. For example in line 320-323 it is not clear if this is symptoms or side effects (while in the next sentence you specific by referring to the pink lines in the figure).

This section of the Results falls under the subheading “Hypothesis 2: Association between infection intensity and symptoms”, which focuses on symptoms. We have added a clarifying term to the beginning of the first sentence to be sure it is clear to the reader (line 371).

Conclusions:

The authors conclude that co-infections and other sources of morbidity may influence the reporting of symptoms and side effects. Could the authors clarify how this conclusion was drawn, given that other infections or causes of morbidity do not appear to have been directly assessed in the study?

We thank the reviewer for this thoughtful comment and the opportunity to clarify. It is correct that our dataset did not include information on co-infections or other potential sources of morbidity, and we therefore did not conduct any formal analyses on these factors.

However, in the Discussion (lines 525-541), we explored the possible influence of co-infections and other local disease burdens on the reporting of symptoms and side effects. This was based on contextual information and literature, including:

- Reports that Bugoto LV had undergone MDA with albendazole in 2003, and that symptoms of STH can overlap with those of intestinal schistosomiasis.
- Documentation on the water quality and health risks around Lake Victoria, where diarrhoeal illnesses are common.
- The known overlap between malaria- and schistosomiasis-endemic regions, and the similarity in symptoms such as headache, diarrhoea, and nausea.

These references are included in the Discussion (lines 525-541).

We agree that our previous wording in the Abstract's Conclusion (lines 47-51) may have led to misinterpretation and have now revised the sentence accordingly.

Summary and General Comments:

Since the study focused on school-aged children, I was curious to what extent children actually refuse praziquantel during MDA. The study from Adriko et al (reference 38) concludes that being enrolled in a school actually improves the odds of taking praziquantel. Is the study population in your study representative, or should praziquantel uptake also be investigated in adults? Is treatment refusal more commonly seen among adults in the community?

This is a valuable point, and we thank the reviewer for pointing out that we have not used the most robust literature to evidence our conclusion.

Whilst Adriko et al does show that being enrolled in school increases MDA uptake, the reality is that a significant proportion of East African children are not enrolled in school (~30% according to UNICEF). The review by Burnim, Ivy & King (PLoS Negl Trop Dis, 2017) highlights that school-based deworming programmes have the lowest coverage of children both enrolled and non-enrolled. They also specifically highlight that the fear of side effects substantially reduces uptake – in particular, one study saw only 28% of eligible children taking MDA, and 72% of the refusals were due to a fear of side effects.

They additionally make the point that a better understanding of schistosomiasis and the treatment improved MDA uptake. This is in line with our broader conclusion, that findings like ours are key to producing evidence-based educational materials that could improve MDA uptake.

We have therefore updated the text to reflect this use of a more suitable evidence source and improved the phrasing of our point to be more reflective of where we are drawing these conclusions from. Please see lines 462-464.

While the study offers interesting insights, I am concerned about the relevance of the findings given that the data were collected over 20 years ago (2004). The manuscript does not discuss how the epidemiological context may have changed since then, particularly in terms of prevalence, intensity of infection, and overall health status of affected populations. The authors should address this limitation in the discussion, and explain how their findings can be interpreted in light of current MDA practices and transmission dynamics, and to discuss the limitations of applying results from such historical data to today's settings.

We understand the reviewers concern about relevance given the time passed since these data were collected. However, we believe that these data and findings are unfortunately still highly relevant. Studies conducted in the same communities from 2017 to 2018 have shown that prevalence has not significantly reduced over the intervening 13-14 years of treatment, with prevalences of 99% (95% CI: 97% - 100%) in SAC across these three schools. and that the impact of treatment is short-lived suggesting a substantial reservoir of infectious material in the environment (Clark *et al*, clin infect dis, 2022). Additionally, Sergi *et al*. (Proc R Soc Lond B Biol Sci, 2024) reported *S. mansoni* prevalence in Mayuge District was 60.64% in SAC, with 20.24% of infections classified as heavy, which similar to our 2004 estimate of 21% with heavy infections within the villages. Furthermore, malaria is also still highly prevalent and STH still prevalent, albeit still at a low level. Health system infrastructures have improved but not greatly, and Musubi still does not have a health centre. We have updated text on lines 662-670 to reflect that we have considered the timeframe of this study but still believe our findings to be relevant.

How accurate are the high(er) egg counts, eg above 500 EPG? It would be good to also mention the infection intensity categories based on KK. For example the mean EPG Musubi CoG is >400 indicating heavy infection intensity, while the mean EPG for Bugoto is ~100 indicating moderate infection intensity. Since the infection intensity (EPG) is most likely not normally distributed, I would be more interested in the median EPG and the range. Is the median EPG per school showing the same?

We thank the reviewer for this helpful suggestion. In response, we have replaced the mean ± standard deviation with the median and interquartile range (IQR) for EPG values in Table 1 to better reflect the underlying distribution. In addition, we have now categorised infection intensities using the WHO Kato-Katz thresholds (light, moderate, heavy) and included this information in Table 1 for clarity.

The study uses pre-treatment reported symptoms in the analysis, but in the results the authors do not specify whether these pre-treatment symptoms are present at the moment or if they had experienced these symptoms in the past. I would be more interested to see the analysis focused on ongoing symptoms and whether this was associated with infection probability.

Our symptom data reflect whether participants had ever experienced these symptoms, rather than whether they were ongoing at the time of data collection. Given that schistosomiasis is a chronic infection, we consider “ever experienced” symptoms to be a reasonable proxy for ongoing disease manifestations. Chronic infections such as *S. mansoni* may lead to intermittent or recurrent symptoms, and thus previously experienced symptoms may still be reflective of current morbidity and we have clarified this in the text. Please see lines 229-231 with text to this effect. Our dataset does not allow us to analyse ongoing symptoms from treatment side effects. We have therefore acknowledged this in the revised manuscript as a limitation (see Limitations section, lines 593-608). Specifically, we now state that further research examining the impact of underlying symptoms on side effect reporting would help clarify whether such an association exists.

Reviewer 2’s Comments:
Methods:

•Line 157: what was the intended sample size and calculation? The actual number sampled and why the sample size was not met should be covered in the Results section.

We thank the reviewer for raising this point. Whilst the sample sizes in our final dataset are indeed small, we did not state that the required sample size was not met. These analyses were undertaken retrospectively using the available data, rather than as part of a newly planned, power-calculated study. Importantly, we have employed Bayesian analytical methods, which are substantially more sensitive than a frequentist approach, thereby yielding greater power to handle smaller sample sizes. We therefore do not believe that our findings have been materially affected by the available sample sizes. Furthermore, we restricted our analyses to individuals with both Kato-Katz and POC-CCA data available. Whilst this inevitably reduced the number of observations, and thereby statistical power, it substantially improved the accuracy of infection status estimation, thereby enhancing the reliability of our findings.

•Line 162: If not included in analysis then don’t include in the methods

Thank you for the suggestion. This section has now been removed from the Methods as the data were not included in the analysis.

•Line 170: Was urine collected and tested for POC-CCA in these sites 2004?

Yes, POC-CCA testing was conducted in 2004 using fresh urine samples collected at each site. This has now been clarified in the Methods section, please see line 204-205.

•Line 172: This implies the sample children received PZQ following the study as well as PZQ through the national MDA programme (lines 150 – 152)?

We thank the reviewer for highlighting this important clarification. We have amended the text (lines175 – 180) to state that treatments were administered and directly observed by the study team, rather than by the national programme. We also note that children may have subsequently received praziquantel during the routine national MDA campaign later in the same year.

•Line 177: Participants with > 100 epg at 1 week received a second praziquantel dose – there is no mention of how this alters the four-week clearance probabilities and whether analyses stratified by single vs. double dosing are feasible. The Limitations section downplays the importance of this key factor which is likely significantly skewing the results of the study (line 548)

Thank you for raising this point. We have added lines 217-226 to the methods to direct the reader to the limitations section, frontloading this issue. We have also updated the text in the limitations section. The text we have added makes the point that that the provision of a second treatment, only impacts the findings related to hypothesis 5 and not hypotheses 1-4 at all. Data collected on pre-treatment symptoms, the side effects, and infection intensities/ prevalence pre-treatment were all collected prior to any second treatments. We likely somewhat over-estimate clearance and have made this clearer in the main text. We have also made an additional Figure S4, which shows that second treatments do not lead to a 100% probability of clearance either, which suggests we are not necessarily over-estimating clearance by as much as we think.

•Line 182: Need to say why the one-week post-treatment data not included.

We have improved our explanation of this in the updated methods section. Please see lines 216-219.

•Line 192: S. haematobium

We thank the reviewer for pointing this out. As this is the first mention of *Schistosoma haematobium* in the manuscript, we have retained the full name. This has now been corrected in the text. Please see line 233.

•Line 219: Trace as positive or negative?

This model framework was developed to overcome this issue. None of the raw POC-CCA scores are allocated a positive or negative score. Instead, each score is assigned a numeric score from 0 (negative), 1 (trace), 2 (+), 3 (++) to 4 (+++). The observation process of the LCA models infection intensity in terms of eggs within an individual, which is linked to the likelihood for the POC-CCA scores via a logistic function, where the numerator is the maximum score possible and the real number of the denominator is the egg counts estimated by the observation process of the model, forming a hierarchical structure of estimation. This only makes the assumption that as the egg counts increase, the POC-CCA score will increase, which we believe to be a reasonable assumption and is described in the cited references.

Results:

•Line 281: we know this from the Methodology section

This sentence has now been deleted.

•Line 282: we have no idea whether the number of children sampled is sufficient to perform the analysis. Why were there nearly twice as many participants for Bugoto than for Musubi?

This was the number of children who provided a full set of data and were enrolled in the programme from each school. As this analysis was conducted retrospectively on data that were collected in 2004, there was no scope to alter these numbers. We have chosen not to perform post-hoc power analyses as these are highly problematic, as supported by a large body of published literature. However, as noted elsewhere, the use of Bayesian methodology here mitigate the issue of small sample sizes. Additionally, as the analysis is not stratified by age, sex etc., the sample sizes are not insubstantial.

•Line 282: These are not final numbers in the S1 Table

We thank the reviewer for pointing this out. We have now added the relevant clarification in the manuscript. Please see lines 185-190.

•Line 284: what are these values? Assume eggs per gram?

In line with Reviewer 1’s suggestion, we have replaced these values with infection intensity data presented as median [Interquartile range]. These values are expressed in eggs per gram (EPG), and we have clarified this in the manuscript. Please see lines 330-332.

•Line 286-287: This are not the same figures as in Table 1?

We thank the reviewer for pointing this out. We have revised the manuscript accordingly.

•Line 290: Figures are not same as in table.

We have checked the figures and revised the manuscript accordingly.

Table 1- Please can the authors review and ensure everything in the table is explained i.e. what are the percentages?; where are the POC-CCA results?
Tables / Figures - too many – which are the key ones

Please find the tables updated to reflect these requests.

Each table within the results section provides the results of the analysis that was conducted. It was concluded that reporting this in the text would be more verbose than the tables. As the values presented in tables 2-6 are the regression results these are key and are supported by the figures.

Conclusions:

•The conclusions are supported by the data presented however statements about symptoms resolving at lower intensity thresholds overstate what your four-week follow-up can demonstrate, given the sparse data in the highest-intensity categories

Sorry for any confusion here, but we do not draw the conclusion that the symptoms are resolved at low infection intensities – we show that there is a non-linear relationship between some symptoms and infection intensity, whilst others show no relationship. There is a substantial amount of discussion given to the small sample sizes in the highest infection intensity categories. Whilst there are indeed very few children in the highest infection intensity categories, were there an indication of a strong positive relationship, this would presumably be observable.

•Limitation - small sample sizes at high intensity levels and the binary nature of symptom reporting are acknowledged but there also needs to be mention of potential semi-quantitative misclassification in the POC-CCA scoring ("Trace" vs. "+") and the absence of longer-term (beyond four weeks) efficacy data.

As described in detail above and in the manuscript (lines 261-273) we make no assumptions about the interpretation of POC-CCA scores and positivity but use the 'probability of infection' as derived from the latent process model.

•A brief caveat about unmeasured confounders (co-infections, immunity status) should be included in the limitations.

This is now addressed in the Discussion (see lines 525-541 and 561-568).

Summary and General Comments:

The manuscript needs to be re-read and significantly edited by the first and senior authors to ensure it meets publishing quality, there are quite a few inconsistencies in terms of side effects and side-effects; minor mis-spellings (there are a lot of extra 's' on words), consistent order of narrative e.g side-effects and symptoms vs symptoms and side-effects, the names of the schools; overuse of the word hypotheses/hypothesises etc. The manuscript comes across as a student project dissertation rather than a publication ready for peer-review.

We have now carefully re-read and edited the manuscript, with particular attention from both the first and senior authors, to address all noted inconsistencies and typographical issues. We also have gone through the manuscript carefully to ensure that we have written side effects and side-effects correctly in each location, including the hyphen for side-effect data, side-effect analyses etc. but not when it is used as stand alone.

The manuscript is relatively long and the methodology, results and discussion sections are overly verbose and need a major rewrite to be made more succinct.
Whilst there are five distinct sets of analysis conducted, which we appreciate is a lot of content, we have strived to reduce the word count and streamline our discussion of each analysis.

There is also no mention of the sample size and how it was calculated.

Please see response above regarding sample sizes.

There are many occasions where the numbers in narrative don't match those in the tables/figures and information also missing.
There are specific comments for each section up until Results where it became clear a major revision of the manuscript was required as the narrative and tables/figures didn't share the same information.

We apologise and thank the reviewer for highlighting these inconsistencies. We have now thoroughly reviewed and revised the manuscript to ensure that all narrative descriptions are fully aligned with the corresponding tables and figures, and that any missing information has been addressed.

Specific comments for other sections
Abstract
•Line 24: use words to describe 'heavily/infected' people – clarify if this means infected people and heavily infected people or heavily infected people?

We have revised the sentence at line 24 to clarify the intended meaning.

•Lines 33 – 39: Principal Findings – I read this a few times and wasn't clear on what

it was trying to communicate and how it linked to the Conclusion section. Rewrite to be clearer.

We have re-written this to be clearer.

•Lines 43-44: its not clear what would be supporting informed discussions with communities or what is inferred by that. Suggest removing or making clearer.

We have removed this sentence.

Author summary

•Line 55: 'discomforts' is very subjective – change to be more specific

This has now been removed.

•Line 58: It is unclear what has been said previously will improive community engagement? There is a large leap between resercah studies determining something and community engagement – need to be clearer.

Our intention was not to imply a direct or immediate link between our findings and increased community engagement, but rather to suggest that a better understanding of how symptoms and side effects are perceived, and their relationships with infection, could inform the development of more tailored health communication strategies. These, in turn, may help address misconceptions or concerns that affect participation in MDA programmes. The improvement of health messaging has been shown to improve treatment update in other studies (i.e. Kim *et al* (BMC public health, 2024)). We have revised the sentence to clarify this point. Please see lines 67-71.

Introduction

•Line 60: change to elimination as a public health problem goals

Thank you for the suggestion. We have revised the sentence (now in line 70) as recommended.

•Line 69: remove 'warm' or replace with specific temperature

This has now been removed.

•Line 73: inaccessible by what/whom? Directly unobservable by what/whom? Re-write to be clearer.

We have revised the sentence to clarify that adult worms cannot be observed in situ in order to determine infection status with gold-standard certainty.

•Line 80: Kato-Katz

We have corrected the misspelling.

•Line 80 – 81: Latent class analyses haven't been developed specifically for schistosomiasis diagnostics – sentence needs reworded to be clearer.

We have reworded the sentence to clarify that LCAs have been leveraged in the context of schistosomiasis research to help interpret imperfect diagnostic data, but were not developed for this specific purpose. Please see lines 96-99.

•Line 88: 'This' what? – need to be clear on what 'This' is referring to.

We have revised the sentence to clarify that it is chronic schistosomiasis that has been associated with these health impacts. Please see lines 105-106.

•Line 107, 108, 114: all sentences start very similarly, need to edit to introduces the sentences differently

We have reworded these sentences to improve stylistic variety while preserving the original meaning. Please see lines 130-137.

•Line 118: presence or prevalence?

This sentence is regarding the presence of infection at the individual level – not prevalence as a community-wide measure and we have amended this in the text to clarify this.

•Line 138 - 140: #5 has not been addressed in the Intro – why do the authors hypothesise this?

In the Introduction (lines 128-133), we summarised recent findings suggesting that praziquantel treatment causes a release of antigens from dying adult worms, that may be responsible for side effects such as abdominal pain or diarrhoea. Therefore, we hypothesised that if this is the case, then people who report more side effects may also be more likely to have had high numbers of damaged adult worms and to have cleared their infections.
